# Phenotyping field-state wheat root system architecture for root foraging traits in response to environment×management interactions

**DOI:** 10.1038/s41598-018-20361-w

**Published:** 2018-02-08

**Authors:** Xinxin Chen, Yinian Li, Ruiyin He, Qishuo Ding

**Affiliations:** College of Engineering, Nanjing Agricultural University/Key Laboratory of Intelligent Agricultural Equipment of Jiangsu Province, Nanjing, 210031 China

## Abstract

An important aspect of below-ground crop physiology is its root foraging performance, which is inherently related to root system architecture (RSA). A 2-yr field experiment was conducted and the field-state wheat RSA was phenotyped for root foraging trait (RFT). Four RSA-derived traits, i.e. Root horizontal angle (RHA), axial root expansion volume (AREV), RSA convex hull volume (CHV) and effective volume per unit root length (EVURL), were analyzed for RFTs in response to environment × management interactions. Results showed a dynamical RHA process but without statistical difference both within crop seasons and tillage treatments. AREV increased with root developmental stages, revealing an overall better root performance in the first year. However, tillage treatments did not induce observed difference within both crop seasons. CHV varied drastically from year to year and between tillage treatments, correlating well to the root length, but not with RHA. EVURL was both sensitive to tillage treatments and crop seasons, being a potential indicator for RFT. Above all, tillage effect on RFT was statistically far less than that induced by crop seasons. Pro/E assisted modeling can be used as an effective means for phenotyping integrated, RSA-derived, RFTs for root foraging response to induced environment × management interactions.

## Introduction

The performance of a field crop is governed by the integrated effects of its structural, physiological and genetically mediated traits being quantified as nutrient status, yield potential and foraged soil volume etc. Quantification of field crop traits both requires a large number of scenarios to be considered^1^ and a range of phenotyping tools dedicating to multiple scales and specific organic components of crop responding to the environments. Among which, root foraging capacity depends on root system architecture^2^.

Root system architecture (RSA), the spatial arrangement of the root and its components^3^, is generally described at three distinctive levels, i.e. the topology level, the geometry level and the root segment level^4–6^, serving as an overall transcription of the process of root-soil interplay in the field environment. The topology implicitly describes the root system as a network, or skeleton, of a root system. The geometry of a root system explicitly describes the physical position of component root axes. While the root segments can be characterized by their properties, such as local root diameter, color and the presence or absence of root hairs^7^. Being a key component to water and nutrient acquisition, field crop RSA varies between and within species, and also subject to environment changes^8^. However, ‘this hidden half’ is difficult to interpret^9^.

Implicitly or explicitly, RSA was evaluated at sub-topology level of crop roots, e.g. root length, number, positioning, and angle of root components^3^. However, these sub-topological root quantifications could not effectively explain the overall developmental dynamics and root foraging traits (RFTs). RSA was more reasonably defined as the spatial distribution, age, and identity of all roots from a single plant^10^. Under the light of this definition, RFT should be more preferably related to the foraged soil volume. And thus the phenotyping of root responses to the prevailing environments, a key focus for field crop physiologies, could possibly be made with RSA-derived indices.

Despite the established implications of RFT from root distribution in soil profile and the application of it for quantifying soil heterogeneity^11,12^, it would be more meaningful to monitor RSA-derived indices and to quantify RFT and its interaction with the surrounding environment from the dynamics of RSAs. While the mass-based or length-related indices (e.g., root allocation, root foraging scale, root foraging precision, root foraging rate)^13^, would be less emphasized.

In foraging the soil, field crops implement several well-established developmental plans, e.g. hydrotropism and gravitropism. The synergetic effects from genetic control and environmental modification are integrated into root decisions regarding how fast and in which direction to grow, and where and when to initiate new roots^14^. This ability of a field crop to adjust its RSA is defined as root plasticity^15^, an important strategic trait of plant in coping with the large variety of abiotic conditions in field^16^. Understanding the developmental and architectural plasticity of RSA thus holds great potential for stabilizing productivity under suboptimal environmental conditions^10,17,18^.

Field-based phenotyping of RFTs, or plant phenotyping methods in general, applicable for plant phenomes still lags greatly behind^19^. Despite the expectation of improved productivity gains from optimized RSA^20^, this goal is hampered by the low resolution and low throughput approaches for characterizing crop RSA^10^. Even the extraction of the entire root system from soil could be an impeding factor for field-based phenotyping of RSA^16^. Therefore, a number of efforts were made to transfer phenotyping techniques from controlled environments to in-field phenotyping^19,21,22^.

The aim of this research was thus to investigate what RSA-derived indices could be made from the field-state wheat root systems and how these parameters could be used for quantifying RFT under the governing mechanisms by the environment × management interactions.

## Results

**Figure 1:**
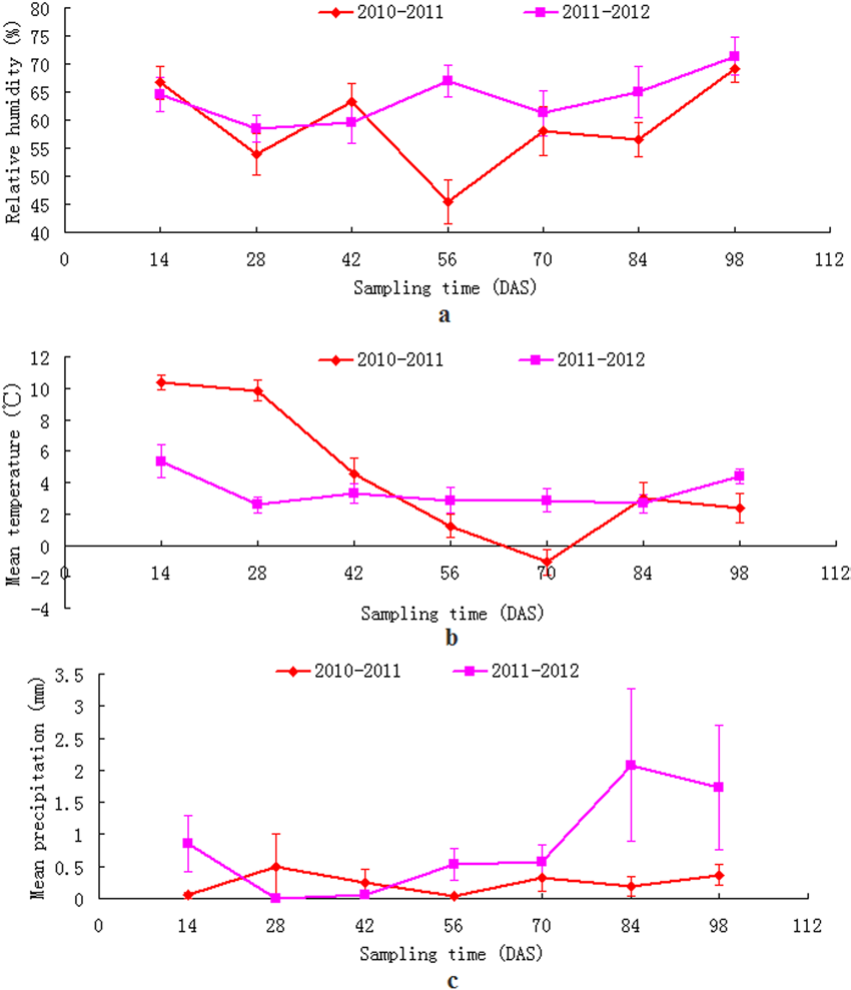
Meteorological processes during wheat growth period.

Largely varied meteorological processes occurred in the two crop seasons (Fig. 1). Overall precipitation and relative humidity in 2010–2011 was higher than 2011–2012, yet the annual mean temperature of the first crop season was less than the second. The stochasticity of meteorological data also implies that no replicative environmental factors could be controlled within the field experiment.

### RHA dynamics

**Figure 2:**
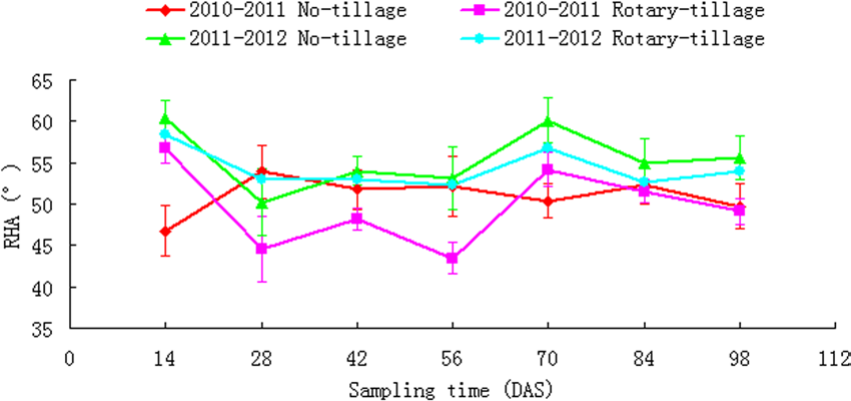
RHA dynamics under 2 tillage systems and in 2 yrs.

Apparent variations of wheat relative horizontal angle (RHA) were observed, in different sampling time, under different tillage treatments and in different crop seasons (Fig. 2). But the deviation of this variation was not large, with a mean of about 52°. Statistical analysis revealed that RHA was neither co-related with RSA-derived indices (AREV, CHV and EVURL) nor with meteorological parameters (MP, RH and MT). While the T-test indicated that inter-annual meteorological difference had no significant influence on RHA of no-till wheat RSA, a marked effect was observed on the rotary tilled (P < 0.01) (Table 2). Within the same year, tillage treatments had no significant effect on RHA.

Despite overall statistical analyses, Fig. 2 also revealed two 2 large variations identified as marked deviation from and with non-overlapped error bar with other subsets, i.e. one was the sinkage in the 56 days after sampling (DAS) of the rotary-tilled wheat in the first crop season and the other a surge in the 70 DAS in the no-till wheat in the second crop season.

### AREV dynamics

**Figure 3:**
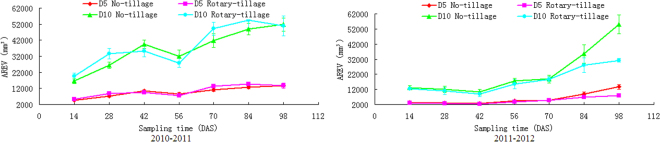
AREV dynamics (D5: axial root expansion with 5 mm, D10: axial root expansion with 10 mm).

Plots of the axial root expansion volume (AREV) indicated an increased soil volume exploited by wheat root with respect to both sampling time and expansion scale (Fig. 3). Increased soil volume foraged by the root may imply an increased potential for water and nutrient absorption. Gahoonia and Nielsen^23^ asserted that increased soil volume for a root system is particularly critical for immobile nutrients. A larger foraged soil volume is generally more preferred than a small one. As thus considered in 2010–2011 overall performance of AREV under rotary till is better than no-till. But in the following year the no-till treatment performed much better. Statistical analysis revealed that, regardless of yearly difference and tillage treatments, AREV was both significantly related to AREV and CHV, with a correlation coefficient no less than 0.89 (P < 0.01) (Table 1). No significant correlation was observed between AREV and meteorological indices (MP, RH and MT). T-test also revealed significant effect of yearly difference on AREV (0.01 < P < 0.05) under no-till, and that was extremely significant (P < 0.01) under rotary tilled condition (Table 2). However, within the same year, tillage treatment had no effect on AREV dynamics (Table 3).

**Table 1:** Correlation coefficients and significant levels of the relations between root foraging traits and environmental factors.

2010–2011		No-tillage							Rotary-tillage			
		RHA	AREV	CHV	EVURL	MP	RH	MT	RHA	AREV	CHV	EVURL
No- tillage	RHA	1	0.268	0.184	0.243	0.448	−0.668	−0.153	−0.789*	—	—	—
	AREV	0.268	1	0.98**	0.894**	0.302	0.15	−0.731	—	0.924**	—	—
	CHV	0.184	0.98**	1	0.955**	0.178	0.178	−0.785*	—	—	0.866*	—
	EVURL	0.243	0.894**	0.955**	1	0.063	0.011	−0.867*	—	—	—	0.699
Rotary-tillage	RHA	−0.789*	—	—	—	−0.233	0.61	0.063	1	0.083	0.067	−0.133
	AREV	—	0.924**	—	—	0.464	0.138	−0.629	0.083	1	0.988**	0.954**
	CHV	—	—	0.866*	—	0.477	0.23	−0.565	0.067	0.988**	1	0.946**
	EVURL	—	—	—	0.699	−0.67	0.07	−0.54	−0.133	0.954**	0.946**	1
	MP	0.448	0.302	0.178	0.063	1	—	—	−0.233	0.464	0.477	−0.67
	RH	−0.668	0.15	0.178	0.011	—	1	—	0.61	0.138	0.23	0.07
	MT	−0.153	−0.731	−0.785*	−0.867*	—	—	1	0.063	−0.629	−0.565	−0.54
**2011–2012**		**No-tillage**							**Rotary-tillage**			
		**RHA**	**AREV**	**CHV**	**EVURL**	**MP**	**RH**	**MT**	**RHA**	**AREV**	**CHV**	**EVURL**
No- tillage	RHA	1	0.056	−0.011	−0.008	0.373	0.494	−0.176	0.882**	—	—	—
	AREV	0.056	1	0.988**	0.941**	0.19	0.375	−0.386	—	0.954**	—	—
	CHV	−0.011	0.988**	1	0.963**	0.183	0.377	−0.375	—	—	0.936**	—
	EVURL	−0.008	0.941**	0.963**	1	0.049	0.187	−0.522	—	—	—	0.962**
Rotary-tillage	RHA	0.882**	—	—	—	−0.192	0.498	0.21	1	−0.115	−0.223	−0.268
	AREV	—	0.954**	—	—	0.112	0.236	−0.491	−0.115	1	0.974**	0.972**
	CHV	—	—	0.936**	—	0.145	0.237	−0.427	−0.223	0.974**	1	0.987**
	EVURL	—	—	—	0.962**	0.106	0.086	−0.501	−0.268	0.972**	0.987**	1
	MP	0.373	0.19	0.183	0.049	1	—	—	−0.192	0.112	0.145	0.106
	RH	0.494	0.375	0.377	0.187	—	1	—	0.498	0.236	0.237	0.086
	MT	−0.176	−0.386	−0.375	−0.522	—	—	1	0.21	−0.491	−0.427	−0.501

**Correlation is significant at the 0.01 level (2-tailed), *Correlation is significant at the 0.05 level (2-tailed), the data means Pearson correlation. Radius of AREV was 5 mm.

**Table 2:** T-test of RFTs in different years.

	No-tillage				Rotary-tillage			
	RHA	AREV	CHV	EVURL	RHA	AREV	CHV	EVURL
Abs(t)	2.061	3.255*	4.545**	4.679**	3.886**	6.552**	5.538**	8.345**
Sig	0.085	0.017	0.004	0.003	0.008	0.001	0.001	0

**Difference is significant at the 0.01 level (2-tailed), *Correlation is significant at the 0.05 level (2-tailed). Radius of AREV was 5 mm.

**Table 3:** T-test of RFTs in different tillage.

	2010–2011				2011–2012			
	RHA	AREV	CHV	EVURL	RHA	AREV	CHV	EVURL
Abs(t)	0.505	1.306	0.567	0.904	1.63	1.605	1.802	2.302
Sig	0.632	0.34	0.591	0.401	0.154	0.16	0.122	0.061

**Difference is significant at the 0.01 level (2-tailed), *Correlation is significant at the 0.05 level (2-tailed). Radius of AREV was 5 mm.

### CHV dynamics

**Figure 4:**
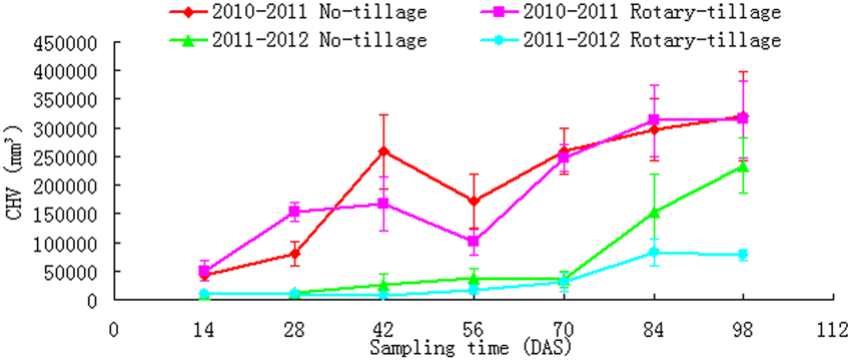
CHV dynamics in 2 tillage treatments and in 2 yrs.

RSA enveloped soil volume (CHV) varied dramatically from year to year and between different tillage treatments (Fig. 4). The 2010–2011 wheat seasons revealed an overall higher value in comparison with the next crop season, indicating that a 15 d earlier sowing of wheat could have significant influence on RSA performance during the whole crop seasons. In the 56 DAS of the first wheat season there was a radical drop of CHV, both for no-till and rotary-till. This is in contrast with the next wheat season, when the CHV increased moderately.

Disregarding RHA and irrespective of yearly difference and tillage methods, CHV was found significantly correlated with AREV and EVURL and with correlation coefficients higher than 0.93 (P < 0.01) (Table 1). CHV had no correlationship with meteorological indices (MP, RH and MT), with only one exception in the no-till in 2010–2011 when it was significantly related with MT (correlation coefficient −0.785) (0.01 < P < 0.05). T-test revealed remarkable influence of yearly difference on CHV (P < 0.01), in both the two tillage treatments (Table 2). Whereas tillage methods within a year had no significant influence on CHV (Table 3).

### EVURL dynamics

**Figure 5:**
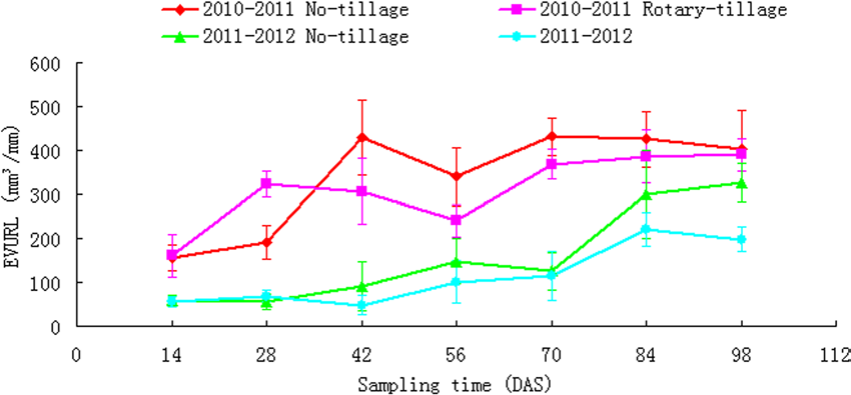
EVURL dynamics under 2 tillage systems and in 2 yrs.

Unlike RSA dynamics, the appearances of effective volume per unit root length (EVURL) clearly differentiated root-soil interaction in both tillage treatments and crop years (Fig. 5). Not only the first year had remarkable higher EVURL values than the next year, but also the no-till EVURL out-performed the rotary-till. This means that EVURL may be a sensitive RSA parameter, which can be used for differentiating a wider range of environmental factors on wheat RSA. However, even though with these differences, tillage effect on EVURL is far less than the crop seasons (Tables 2 and 3).

## Discussion

### Root foraging traits in response to environment×management interactions

A plant phenotype in a specific agricultural system is considered as the result of the complex genetype × environment × management interactions^20,24,25^. However, unraveling these interactions is not only constrained by the time-consuming, labor intensive, and generally destructively operated approaches^26^, but also constrained by the poor control of the boundary conditions for field-based phenotyping of RFTs. Observed correlations among RSA-derived RFTs (Table 1, Fig. 1) indicated that there is possibility to discriminate environment × management induced RFTs with even basic controls, e.g. seasonal variation and tillage treatments. Disregarding the genetic variations, RSA-derived RFTs of wheat were both affected by alternative seasonal climate (Fig. 1, Figs 3–5) and soil management practices (soil tillage). These variations also imply that quantification of field-state crop RSA plasticity is possible with parameterized RSA-derived traits (Table 1). EVURL could particularly be applied as a stable indicator for RSA-related foraging traits in responding to the tillage system. But for yearly differences remarkable differences were observed among them (P < 0.01) (Table 2). However, as no genetic variation was made in this experiment, observed correlations of RFTs to the environment × management interactions may subject to changes.

Although phenotyping the field crop is becoming a focus of the crop research, field-based phenotyping is largely subjected to dispute. As not only the interactions among plant genomics, field environment, soil and crop management complicated the experimental design in field, but also in general the objective of a field-based phenotyping task could be inconspicuous, due mainly to a poor definition on target traits. Some researchers even stated that phenotyping for field crops could never be possibly made, because the plant phenotype is infinite, vary morphologically and molecularly over developmental time and in response to the environment^27^. The more we examine roots, the more complex their responses and interactions prove to be^28^.

The results of our work, however, supplied some insights on the root foraging responses to the induced changes of system boundary conditions. For instance AREV in the first year experienced an apparent sinkage corresponding to the 56 d period (Fig. 3), which was haply coincided with a minimum mean relative humidity, i.e. the highest degree of dry air (Fig. 1a), a period of relative low temperature (Fig. 1b) and the minimum level of periodic precipitation (Fig. 1c). In the second year, however, a local noticeable dome of AREV appeared in the 56 d period, which was also haply coincided with a period of domed mean relative humidity (Fig. 1a) and a local increase of precipitation (Fig. 1c). Past observations had revealed that differences in soil temperature and water regimes influenced root distribution in the field, as root growth was affected by both soil temperature and soil water content^29,30^. Furthermore, soil temperature and soil moisture were both related to air temperature and precipitation^31^. Unfortunately, systematic sampling of soil-related parameters were not considered in this experiment, leaving the chain effects among these interrelations unanswered.

Inter-annual comparison on CHV dynamics revealed an overall higher value in the first year as compared with the second (Fig. 4). This can be accounted for with an obvious high temperature in the initial 42 d period in the first yr when compared with the second yr (Fig. 1b). Also, mean precipitation in this period was also higher than the second yr (Fig. 1c). The overall higher value of CHV in the first yr indicated that, once an initial superiority of RSA development has been established, crop could withhold its superior soil exploitation performance also in its later developmental stages. Identified RFT correspondences to the induced changes of both soil and the environment indicated that field-based phenotyping could be applied as a means to interpret RSA responses to genetype × environment × management interactions in field.

### Phenotyping the integrative traits for root foraging performance

RSA is generally defined as the spatial arrangement of the root and its components^3^, the quantification of which was generally made with sub-root system parameterizations (e.g., root angle or root length)^32,33^. In its strict sense, these ‘low-level’ parameters could not be used as the overall performance of an entire root system. Assembling the organic and tissue level parameters as a whole is therefore an important measure for ‘integrative traits’. However, these RSA level integrative traits are difficult to be assessed with traditional root research methods (e.g. soil coring). Modelling-assisted RSA analyzing provides a means to quantify root-system level foraging performances.

Recent advancement of RSA-related phenome research has promoted the provision of a number of modern tools, many of which were resorted to computer science or image processing, e.g. DART^34^, SmartRoot^35^, RootNav^36^, RootTrace^37^, RhizoScan^38^, RootSystemAnalyser^39^. As these platforms are largely varied from one to another, cross-platform protocols are needed, paving a way for inter-platform exchanges of information, e.g. archiDART package^40^ and RSML package^6^. However, most of these RSA trait-analyzing platforms were still used for sub-root system level parameters, i.e. geometrical or segmental level indices.

The difference between an analytical, sub-root system trait and integrated, RSA-derived ones could be illustrated with a comparison of RHA responses to environment × management interactions with that of others. Root angle has long been used as a principal component of RSA, being strongly associated with resource acquisition efficiency^41^. RHA-related foraging traits are primarily governed by plagiogravitropism^42^. It is also affected by soil temperature^43^, soil water status^44^, and levels of phosphorus^45^, nitrogen^46^ and soil strength^42^. In this study, however, RHA did not significantly vary under induced treatments, indicating the importance of integrative traits as compared with sub-system ones. Integrated traits of plants are thus more meaningful as they provide synthetic information about interactions between plant organisms and the environment^47^. The plant or even the population level traits explain the overall functional aspects of the root system^5^.

As the field-state plant growth is largely irreversible and root morphology and topology encodes a ‘morphological memory’^48^, RSA-derived traits provide us a cue for the interpretation of plant interactions with its environment. However, as the potentially useful crop traits are enormous, it could be a problem as how to support and counter the relative importance of each trait^11,28^. Nielsen *et al*.^49^ advocated the use of architectural models for integrated root performances. Our results provided a strong support to his statement, showing that Pro/E assisted modeling for RFT analyzing provides an effective means for the study of field crop physiology. The two ‘virtual’ foraging schemes implemented with Pro/E modeling, i.e. axial root expansion and RSA enveloping, quantified the dynamics of soil volume colonization by the crop root. Berntson^50^ used the term ‘potential’ to quantify the ability of a root system in colonizing the soil volume. Evidently increased root foraging potential with root developmental stages (Figs 3 and 4) agreed well with Fitter’s^51^ opinion that increased soil volume explored by the roots is a reflection of the plant’s adaptive ability to make the best use of unevenly distributed water and nutrients in soils. RSA-related traits provided us some hints about plant root strategies when they are faced with insufficient supply of soil water^52^.

## Materials and Methods

### Site description

Winter wheat (Ningmai13) was grown in a paddy field after rice in Jiangpu Experimental Farm, Nanjing Agricultural University, China. The site was located at 31°98′N, 118°59′E, in subtropical monsoon climate, with an annual rainfall of 1048.6 mm and a mean temperature of 15.8 °C^53^. The rice-wheat rotation is a long-established farming system in the region. The paddy season begins in June and ends up by late November. A month before rice harvesting the field was drained, allowing the soil to shift to dry state for mechanical harvesting^54^, following which the dryland crop season, i.e. wheat or canola, is ensued. Soil organic matter, total N, available N, available P and available K were tested to be 8.24 g∙kg^−1^, 0.97 g·kg^−1^, 12 mg∙kg^−1^, 12.67 mg·kg^−1^ and 11.05 mg∙kg^−1^, respectively. Soil pH, bulk density and water content were 7.6, 1.26 g∙cm^−3^ and 29.3%, respectively.

### Experimental design

A 2 yr field experiment was conducted in the wheat season from 2010 to 2012, in which 2 tillage treatments were compared (no-till and rotary till). Seasonal variance and tillage treatments provided combined environmental conditions for RFTs of wheat. In 2010 wheat was sown in 15 November and in 2011 the wheat was sown in 30 November, both just immediately after rice harvesting. No-till is a typical conservation tillage system and rotary till is the best of the traditional tillage system^55^. The field experiment was laid out in three replications per treatment in a plot size of 5 m × 3 m. Wheat seeds were manually placed uniformly on the soil surface in a 5 cm × 5 cm^2^ grid pattern to mimic surface broadcasting, a practice of no-till seeding wheat adopted in some regions of the rice-wheat rotation, e.g. Sichuan, China^56^. The uniform placement of seeds also guarantees a minimised plant-to-plant interaction, which has potential effects on wheat RSA. Seeded plots were covered with a thin layer of fine soil. Phosphate, urea and potassium chloride were applied to the soil surface in an amount of 375 kg∙hm^−2^, 90 kg∙hm^−2^ and 375 kg∙hm^−2^, respectively. The whole wheat season was rain-fed, and the crop was managed in the same way as the local farmers do.

### Root-zone soil sampling and measurement

Root soil was sampled on 14, 28, 42, 56, 70, 84 and 98 days after sowing (DAS). Two plant roots per plot (totally six plant roots per treatment) were excavated and collected in each sampling period^57^. Only those plants with similar height, stem diameter and ear height in the field were sampled and analyzed^58^.

**Figure 6:**
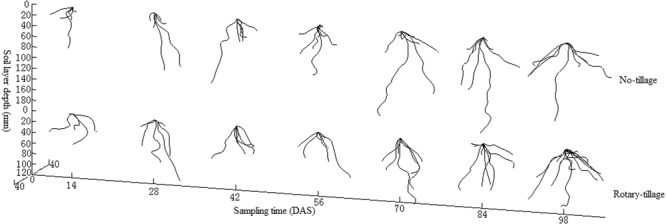
3 D presentation of wheat RSA under two tillage treatments.

A large soil core in 16 cm diameter and 25 cm height was positioned concentric to the base of the plant stem, and driven into the soil with a hand hammer. The core with the soil and the undisturbed wheat root system was excavated with a shovel and was brought to the laboratory for digitizing. Digitizing of wheat RSA was performed with an adapted digitizer. Laminated soil excavation (with 5 mm thickness in each layer) was performed and the exposed root segments in each layer were digitized, providing a set of polar coordinates, i.e. the radius *r*, the angle *φ* and the depth *Z*_*i*_. The collected data was then transferred to the Pro/E for modelling, where reconstructed virtual RSA models were analysed and parameterized^59^ (see Fig. 6). The released version of the software was Pro/E 2.0^60^.

### Root-soil parameterization with embedded algorithms in Pro/E

RSA-derived RFTs were further analysed using the algorithms embedded in the Pro/E tool box. These tools were conveniently used to calculate relative horizontal angle (RHA), axial root expansion volume (AREV), RSA convex hull volume (CHV) and effective volume per unit root length (EVURL).

### Root horizontal angle

The root length or volume was computed as the sum of length or volume of all its segments. Vectors representing each segment of a root were summed up; the angle of this sum toward the horizontal plane was used as the’mean root angle’^61^, which was used for the direction relative to the horizontal plane and measured downwards.

**Figure 7:**
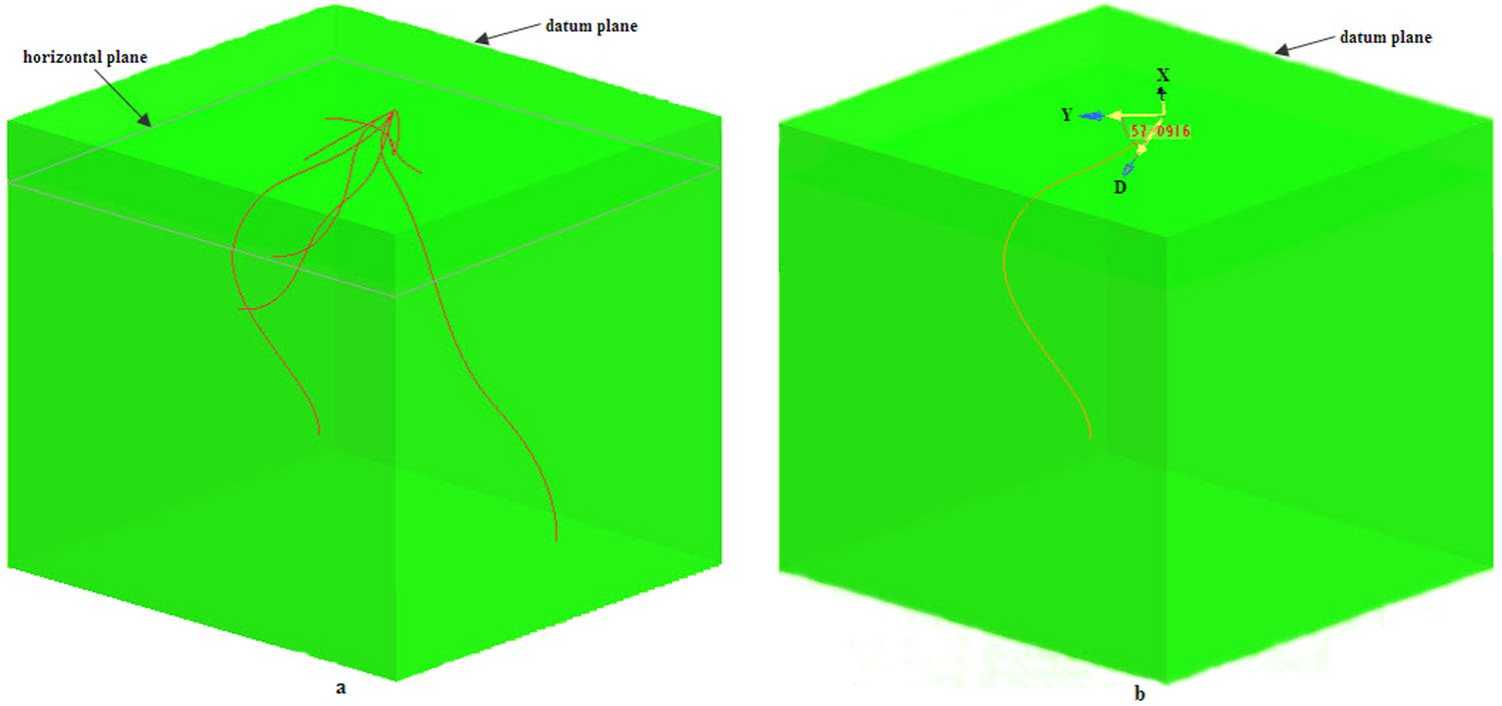
Measurement of root angle in Pro/E.

RHA is the orientation of a root section with respective to the horizontal plane^62^. Pro/E provides a means to calculate this angle by measuring the orientation of a root section with reference to a horizontal plane (Fig. 7a). The horizontal plane containing the datum point (i.e. the seed point) was designated as a datum plane standing for the soil surface. With respect to the datum plane, a sequence of parallel reference planes were generated, each aligning along Z axis and in 10 mm distance from its neighboring ones. These planes intercepted with the virtual RSA, resulting into a series of root sections within each layer. RHA was calculated as the intersection angle between the tangent line D of the root section and the reference plane (determined by the two orientations of X and Y) in each layer (Fig. 7b). The computation was executed automatically in Pro/E with a few commanding steps (i.e., Aanlysis → Measure → Angle), followed by choosing the target root and reference plane and then Execute. The mean of all the calculated angles within a layer is the ‘mean RHA’ for this layer and the mean of all layers represents the ‘mean RHA’ of a plant.

### Axial root expansion volume

**Figure 8:**
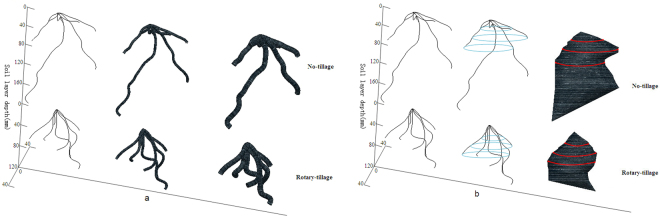
Root-axis-expanded soil models and enveloping soil models.

The mm scale region surrounding crop axial root, i.e. the rhizosphere, is the most active soil volume due to the presence of intense biological activities of both root and microbes. Root suction also induced intense hydraulic gradient within this several mm distance surrounding the root axis^63^. Effective distance for in-mobile nutrient absorption by the root, e.g. phosphate, is limited to 2–4 mm^23^. AREV is calculated from RSA model by expanding the axial roots to a certain diameter, e.g. 5 mm or 10 mm. AREV is thus a surrogate of the effectiveness of soil volume foraged by the crop root system. Pro/E supplies an algorithm to calculate AREV by ‘Variable Cross-section Scanning’. Figure 8a illustrates original wheat RSA and its related AREVs using 5 mm and 10 mm expansion scales. Once the AREVs were generated, its volume can be determined by executing the following Pro/E commands: Analysis → Measure → Volume.

### RSA convex hull volume

RSA convex hull volume (CHV) is the total soil volume exploited by a crop^64^, a parameter indicating the ability of a root system extending in the soil space. Analytical algorithms of Pro/E for CHV is implemented with the following steps: designating the horizontal plane containing the datum point (the seed point) as the datum plane (soil surface), generating a series of parallel planes with reference to the datum plane, being separated 10 mm apart from each other. Each plane intercepts with wheat RSA and results into a number of interceptions, i.e. datum points. The exterior datum points on each plane are used to generate a datum curve, one on each plane. A final stage is to implement a boundary blend tool on all the datum curves, which are merged and solidified to produce the CHV (Fig. 8b). Once the RSA CHV is achieved, Pro/E commands of ‘Analysis → Measure → Volume’ are executed and the volume is calculated.

### Effective volume per unit root length

EVURL is defined as the ratio of RSA enveloped volume to the total root length. A lower value of EVURL indicates that the crop expends less root length for a larger soil volume occupation, thus a higher foraging efficiency.

For all the acquired data, basic statistical analysis was performed and plotted in Microsoft Excel. Correlation analysis and T-test was conducted in SPSS.

### Meteorological data processing

Meteorological data corresponding to the wheat growth stages in the two years was downloaded from the local service webpage of the Weather Online and the data was divided along observation stages. Daily relative humidity, daily temperature and precipitation were averaged as the mean presentation for each stage, being used as referencing basis of the environment for the observed RFTs.
